# Integrating a Large Language Model Into a Socially Assistive Robot in a Hospital Geriatric Unit: Two-Wave Comparative Study on Performance, Engagement, and User Perceptions

**DOI:** 10.2196/81936

**Published:** 2025-12-03

**Authors:** Lauriane Blavette, Sébastien Dacunha, Xavier Alameda-Pineda, Jeanne Cattoni, Anne-Sophie Rigaud, Maribel Pino

**Affiliations:** 1 Institut National de la Santé et de la Recherche Médicale, Optimisation Thérapeutique en Pharmacologie OTEN Unité Mixte de Recherche-Santé 1144 Université Paris Cité Paris France; 2 Service Gériatrie 1&2 Centre Mémoire de Ressources et Recherches Île-de-France-Broca, Assistance Publique – Hôpitaux de Paris Paris, Île-de-France France; 3 Broca Living Lab, Hôpital Broca Paris France; 4 Centre Institut National de Recherche en Informatique et en Automatique de l'Université Grenoble Alpes Grenoble, Auvergne-Rhône-Alpes France

**Keywords:** human-robot interaction, socially assistive robot, older adults, behavioral engagement, multimodal analysis, geriatric care, large language models

## Abstract

**Background:**

Addressing the complex medical and psychosocial needs of older adults is increasingly difficult in resource-limited care settings. In this context, socially assistive robots (SARs) provide support and practical functions such as orientation and information delivery. Integrating large language models (LLMs) into SAR dialogue systems offers opportunities to improve interaction fluency and adaptability. Yet, in real-world use, acceptability also depends on minimizing both technical and conversational errors, ensuring successful user interactions, and adapting to individual user characteristics.

**Objective:**

This study aimed to evaluate the impact of integrating an LLM into a SAR dialogue system in a hospital geriatric unit by (1) comparing system performance and interaction success across 2 experimental waves, (2) examining the links between robot errors, interaction success, and multidimensional user engagement, and (3) exploring how user characteristics influence performance and perceptions of acceptability and usability.

**Methods:**

Over an 8-month period, 28 older adults (≥60 years of age) attending a geriatric day care hospital (Paris, France) participated in a single-session evaluation of a SAR. Interactions took place in the day care hospital and were video-recorded across 2 waves: wave 1 (basic dialogue system) and wave 2 (LLM-based system). From the recordings, system performance (error types and interaction success) and user engagement (verbal, physical, and emotional dimensions) were coded. Acceptability and usability were measured using the Acceptability E-Scale and the System Usability Scale. Sociodemographic data were collected, and quantitative results were supplemented with a thematic analysis of qualitative observations.

**Results:**

Following LLM integration, error-free interactions increased from 27.8% (10/36) to 70.2% (66/94; *P*<.001), comprehension failures decreased from 47.2% (17/36) to 17% (16/94; *P*<.001), and interaction success rose from 25% (9/36) to 74.5% (70/94; *P*<.001). Acceptability (Acceptability E-Scale: 12.8 vs 20.8; *P*=.003) and usability (System Usability Scale: 40.0 vs 60.4; *P*=.04) were significantly higher in wave 2. Engagement scores did not differ significantly between waves, though emotional engagement correlated positively with interaction success (*r*=0.28; *P*=.008), and age was negatively associated with both physical engagement (*r*=–0.30; *P*<.001) and acceptability (*r*=–0.20; *P=*.03).

**Conclusions:**

Behavioral engagement with a SAR in geriatric care is shaped by both system performance and individual user characteristics. Improvements in dialogue quality observed in wave 2, coinciding with the integration of the LLM, were associated with higher interaction success and enhanced user experience. Nevertheless, other contextual or group-related factors may also have contributed to this outcome. These findings highlight the importance of combining multimodal behavioral analysis with self-reported measures to inform the iterative, user-centered design of socially responsive robots in clinical contexts.

## Introduction

The rapid aging of the population worldwide, combined with a shortage of health care professionals, is placing growing pressure on care systems, particularly in geriatric hospitals and long-term care settings [1]. This strain is amplified when caring for older adults with neurocognitive disorders, such as Alzheimer disease and related dementias, whose needs extend beyond medical supervision to include sustained social, emotional, and cognitive support. However, many care institutions, including hospitals, long-term care facilities, and other residential settings, often lack the resources and structures needed to address these complex needs, resulting in care experiences characterized by unmet psychosocial demands, ineffective communication strategies, and increased vulnerability to distress and disorientation [2-4].

Socially assistive robots (SARs) have emerged as a promising technological complement to human-delivered care in such contexts. SARs are designed primarily for social rather than physical interaction, using speech, gesture, and expressive behaviors to engage users and deliver cognitive, emotional, or informational support [5,6]. Building on their social capabilities, SARs have shown potential in enhancing emotional well-being, reducing anxiety and apathy, and promoting social interaction among older adults, particularly in institutional settings, including but not limited to those living with dementia [7-9]. Beyond therapeutic applications, SARs are increasingly explored as tools to assist health care professionals in nonclinical tasks, such as welcoming patients, providing information, or facilitating recreational activities [10-12].

Institutional applications often encompass reception, visitor guidance, and wayfinding. For instance, studies have shown that robots acting as medical receptionists or lobby greeters can facilitate orientation, manage patient flow, and enhance the perceived quality of service, particularly in high-traffic environments [13,14]. A systematic review by González-González et al [15] further suggests that SARs in hospital contexts often adopt hybrid roles, combining informational support with emotional engagement, especially in waiting rooms or ambulatory care settings. Such use cases broaden the vision of SARs beyond therapeutic agents to include communication and orientation facilitators [16], aligning with broader efforts to improve patient experience where staff availability is limited.

However, despite advances in robotics, including improved sensor technologies, natural language processing, and adaptive user interfaces, the integration of SARs into real-world care environments remains limited, with technical limitations representing one of the most significant barriers [17]. Health care providers have reported frustration with issues such as complex operational steps, short battery life, slow system responses, and rigid dialogue systems [18,19]. These limitations may discourage routine use, particularly in dynamic care settings where efficiency is essential. In addition, persistent challenges in personalization and speech recognition further limit deployment, as current systems often fail to accommodate the wide range of capabilities, preferences, and interaction styles found among geriatric care users [20,21].

From the end user’s perspective, errors in human-robot interaction (HRI), including comprehension failures, incoherent or incomplete responses, and misaligned task execution, can erode trust in the robot and reduce engagement [22]. For older adults, even minor conversational breakdowns (eg, misrecognitions or incorrect answers) may trigger confusion, frustration, and diminished trust, particularly when the task is perceived as important [23]. As shown by Kim [24], older adults can also struggle to adapt their speech to the rigid input formats required by conversational systems, sometimes realizing only after delivering a lengthy command that the system had failed to process their request. Such breakdowns not only cause irritation but also expose the gap between the intended “natural” interaction and the constrained, one-directional exchanges these systems often afford. Similar patterns have been observed elsewhere: Khosla et al [25] in a 3-month deployment of a SAR with 5 older adults reported that negative emotions (eg, anger, sadness, and anxiety) occasionally emerged during interactions with the robot, most often when it failed to respond as expected.

Recent advances in large language models (LLMs) offer the potential to mitigate some of these conversational limitations by improving fluency, contextual relevance, and adaptability in robot dialogue systems [26-28]. While early results from other domains (eg, customer service, museum guidance, and educational robotics) are promising, there is limited empirical evidence on how integrating LLMs into SARs affects real-time interactions, error rates, and user experience in real-world geriatric care contexts.

Another critical factor in SAR evaluation is user engagement, which is inherently multidimensional, encompassing verbal behaviors (eg, speech production and turn-taking), physical involvement (eg, gestures and posture), and emotional signals (eg, facial expressions or affective cues) [29]. In HRI, engagement not only serves as a proxy for interaction quality but also predicts subsequent outcomes, such as willingness to re-engage with the robot and the perceived value of the system [30]. Research in care environments, including work with older adults by Hebesberger et al [19], shows that sustained engagement is essential for acceptance and that both technical reliability and interaction fluency shape the depth and duration of participation. Despite its theoretical and practical importance, little is known about how these engagement dimensions are influenced by robot performance, error frequency, or task success in geriatric care contexts, representing a notable gap in the literature.

Finally, user characteristics, including age, socioeducational background, and cognitive functioning, are likely to influence both observable HRI behaviors and subjective evaluations of SARs [31-34]. For instance, older age may be associated with reduced physical expressiveness, while higher cognitive functioning could support more complex conversational exchanges. Understanding these relationships is essential for informing adaptive robot design and deployment strategies that are inclusive and responsive to diverse user needs.

Given these gaps, this study investigated the deployment of a SAR in a hospital geriatric unit, focusing on three main objectives (1) to assess changes in system performance and interaction success following the integration of an LLM into the robot’s dialogue system, comparing 2 experimental waves; (2) to examine the relationships between robot errors, interaction success, and user engagement, considering verbal, physical, and emotional dimensions; and (3) to explore how user characteristics relate to system performance and subjective evaluations of acceptability and usability.

By combining quantitative measures of performance, engagement, and user ratings with qualitative analysis of participant perceptions, this study provides a real-world grounded perspective on the opportunities and challenges of SAR deployment in aging care contexts.

## Methods

### Participants

#### Overview

The study involved older adults attending consultations at the geriatric day care hospital (DCH) of Broca Hospital (Assistance Publique—Hôpitaux de Paris), France. Inclusion criteria were (1) being 60 years and older of age; (2) participating in a scheduled DCH consultation; (3) having a Mini-Mental State Examination (MMSE) [35] score above 10, indicating the absence of severe cognitive impairment; (4) having no current symptoms of altered reality (eg, delusions or hallucinations); and (5) having fluent comprehension and expression in French. No exclusion criteria were applied based on sex, socioeconomic backgrounds, or ethnicity.

#### Recruitment

Recruitment was carried out using the DCH database, and participants were prescreened prior to enrollment, contacted over the phone, and invited to participate in the study the day of their next consultation. An information letter was sent by post, and informed consent was collected onsite.

A total of 41 older adults were recruited for the study. However, this analysis focuses on a subset of 28 participants who met the following criteria: (1) provided consent to be filmed; (2) completed a full interaction session with the robot; and (3) generated usable video data, defined by adequate visibility and recording quality. The remaining participants were excluded due to 1 or more of the following reasons: refusal to engage with the robot, technical malfunctions during the session, or withdrawal of consent for video recording.

#### Setting

The study was conducted between May 2023 and December 2023 in the DCH of a geriatric hospital in Paris (France). The DCH provides specialized outpatient care for older adults with physical or cognitive impairments, offering a wide range of consultations, including neurology, oncology, cardiology, psychiatry, and memory assessments.

The data collection occurred in 2 waves during this period, with distinct participant samples recruited for each phase. All interactions were carried out in a quiet, dedicated room located near the DCH waiting area. This space, typically used for rest and informal activities, was chosen to provide a calm and comfortable environment for testing, while maintaining proximity to the clinical setting.

### Study Design

This cross-sectional observational study was conducted as part of a broader research protocol evaluating the integration of a SAR in geriatric care [36,37]. This analysis focuses on real-world HRI, combining behavioral observations with user-reported data. Each participant engaged in a single, nonscripted interaction session with the robot, followed by questionnaires. Data were collected across 2 experimental waves. The first wave involved a baseline version of the robot without LLM integration, while the second wave used an updated LLM-enabled version. Each wave included a different group of older adults.

### Material

#### ARI Robot

**Figure 1:**
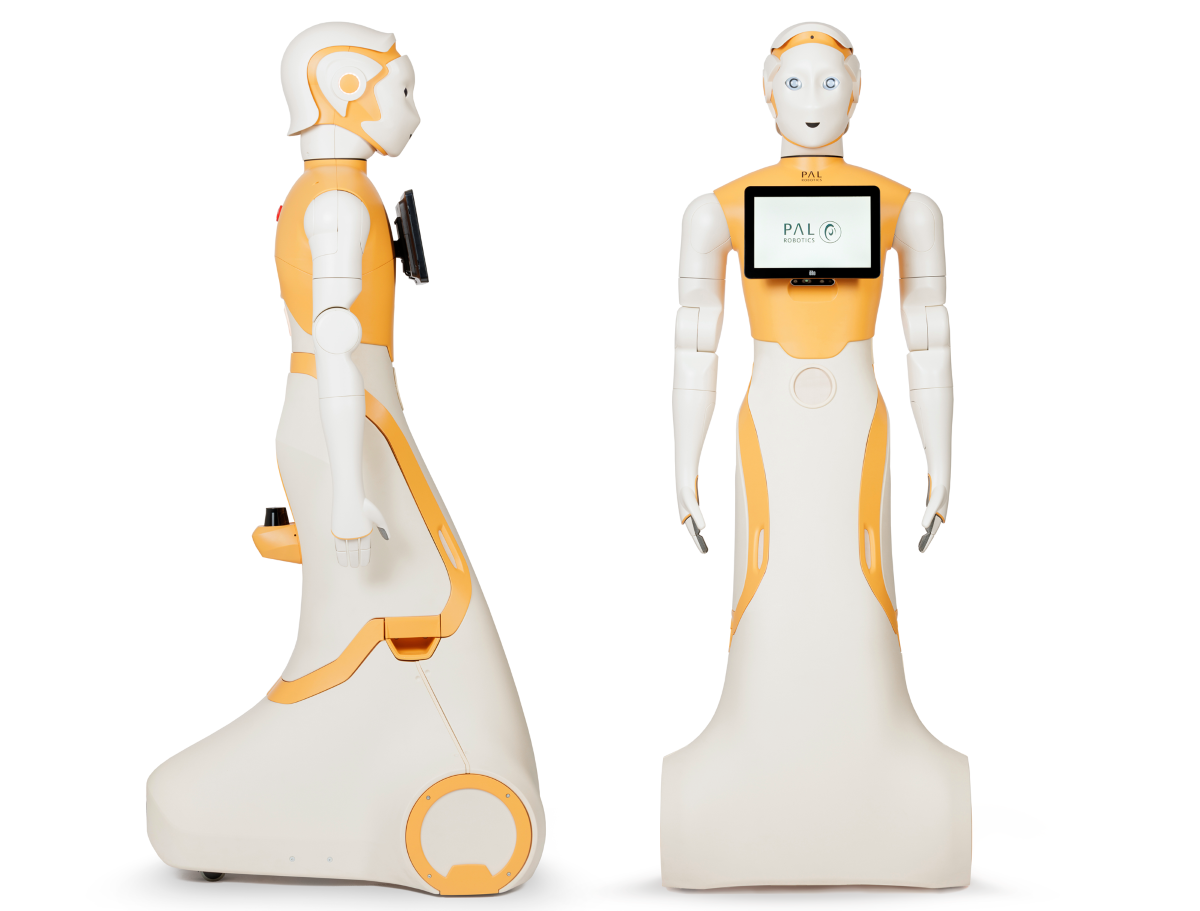
Front and side views of the ARI robot (photo credit: PAL Robotics).

The ARI robot developed by Pal Robotics (Spain) is a 1.65-m (5 ft 5 in) wheeled humanoid platform equipped with a touchscreen, cameras, microphones, animated eyes, and articulated arms (Figure 1). In this study, ARI was used as a socially assistive agent in a geriatric hospital setting.

For this study, ARI was programmed with interaction modules developed as part of the European H2020 SPRING (Socially Pertinent Robots in Gerontological Healthcare) project, aimed at enabling SAR in real-world clinical environments [36]. Two different configurations of ARI’s conversational system were tested during this study, as detailed in the System Evolution section. As ARI had not yet been commercially deployed in care institutions at the time of the study, its use in this context was part of a controlled, exploratory evaluation conducted within the hospital environment.

#### System Evolution

To assess the impact of iterative improvements to ARI’s interaction capabilities, we conducted 2 experimental waves, each following a major system update informed by participant feedback.

- Wave 1 (May to July 2023) deployed a first version of ARI featuring a modular dialogue system based on traditional rule-based intent handling, retrieval-based responses, and basic open-domain generation. This initial architecture was supported by core diagnostic tools, on-screen speech transcription, and perceptual modules (eg, person tracking and facial recognition).
- Wave 2 (September to December 2023) introduced a redesigned conversational module centered on an LLM architecture. Specifically, the system was upgraded to integrate Vicuna-13B-v1.5, a 13-billion parameter LLM optimized for offline use. A custom prompt in French was developed to align with hospital-based use cases, ensuring context-sensitive, coherent interactions. Other system improvements included a refined transcription interface and more efficient vision module processing. All data were securely stored on servers dedicated exclusively to the project. This setup ensured robust data control, as the language model was hosted locally, giving the project partners full control over processing, storage, and use.

A detailed technical overview of these updates is available in SPRING Deliverable D1.6 [38].

#### Assessment Instruments

#### Demographic Data Collection

Basic demographic information was collected using a standardized paper-based form prior to the start of the interaction session. Participants were asked to report their age, sex, and level of education (years of formal education). The level of cognitive function (MMSE) was completed beforehand by the researcher.

#### System Performance

To assess system performance, all interactions were recorded and annotated based on robot behavior. Each interaction was defined as a conversational unit, beginning when the participant initiated a verbal input and ending when the robot responded or failed to do so. For example, if a participant asked, “Where is the restroom?” and the robot replied, “To your right as you exit this room,” the exchange was considered complete, regardless of its duration. Participants could then initiate a new exchange, continue the dialogue, or end the session. This approach produced interactions of widely varying length, from brief question-answer sequences to longer, multiturn conversations, depending on participant intent and conversational flow.

Robot behaviors were annotated using a predefined classification scheme consisting of four categories:

1. Comprehension failure: The robot fails to interpret or meaningfully respond to the user’s input, typically resulting in silence or a fallback message (eg, “I don’t understand”).
2. Inappropriate verbal response: The robot produces a reply that is unrelated, incoherent, or socially inappropriate in context (eg, to the question “What time is my appointment?” the robot replies “Of course. The hospital is open every day from 9 a.m. to 5.30 p.m.”).
3. Incomplete utterances: The robot produces an unfinished or abruptly cutoff response, resulting in a message that lacks necessary information (eg, when asked “Can you tell me where the toilets are?” the robot replies “The toilets are on your left as you come out of ...”).
4. Technical error: System-level malfunctions such as speech synthesis failure, audio dropout, or interface freezing.

The first 3 robot behaviors, comprehension failures, inappropriate verbal responses, and incomplete utterances, were classified as verbal-related errors. This categorization allowed for exploratory correlation analyses between robot behavior and user engagement, enabling a distinction between technical malfunctions and conversational breakdowns.

Multiple error types could be assigned to a single interaction when applicable (eg, a comprehension failure accompanied by a technical issue). This allowed for the analysis of both error frequencies and error co-occurrence patterns as well as comparisons across the 2 experimental waves to examine changes in system performance over time.

#### Interaction Success

**Figure 2:**
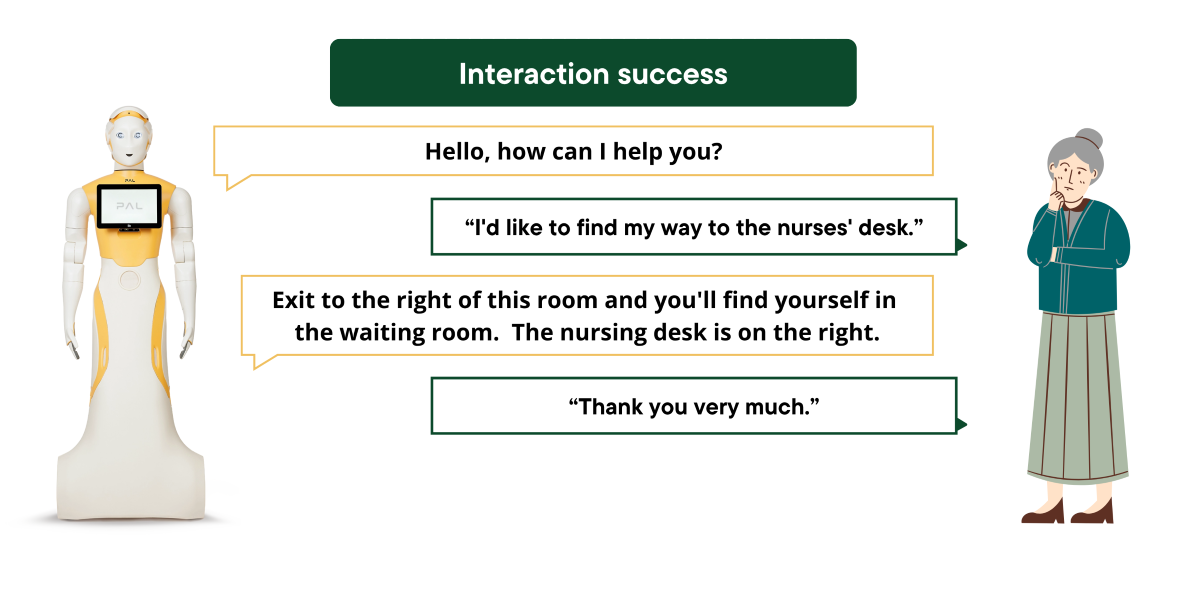
Example of a successful interaction between the ARI robot and a participant.

**Figure 3:**
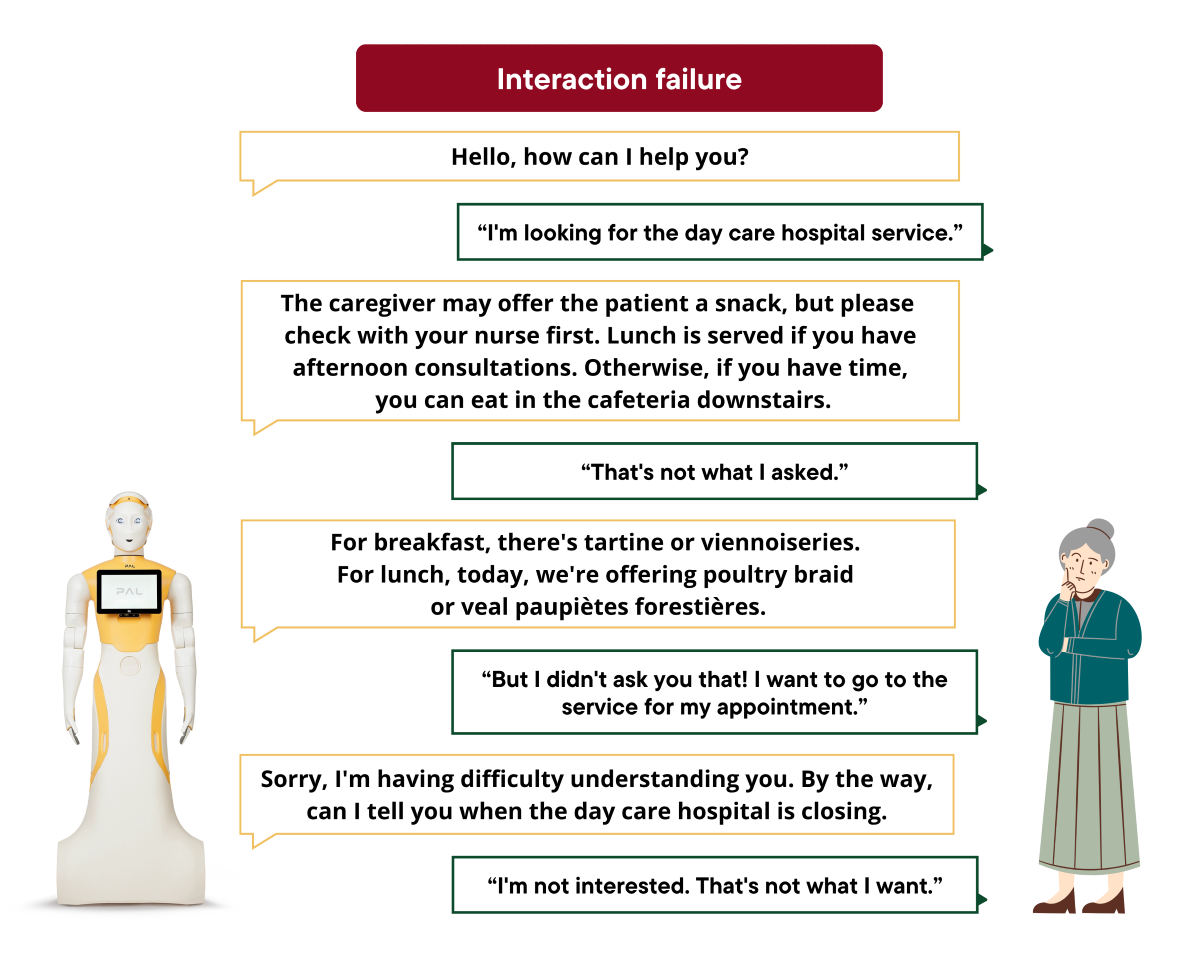
Example of a failed interaction between the ARI robot and a participant.

Interaction success was defined as the robot providing a relevant and coherent response that appropriately addressed the user’s request or corresponded to the information provided. Examples of successful and failed interactions are illustrated in Figures 2 and 3.

#### User Engagement Coding Framework

User engagement was assessed using a structured behavioral coding scheme (Table 1), developed to capture 3 dimensions: verbal, physical, and emotional engagement. The verbal dimension reflected speech production and conversational responsiveness; the physical dimension encompassed posture, gesture use, body orientation, and other forms of physical involvement; and the emotional dimension captured visible emotional expressivity. Each dimension was rated on a 5-point scale, ranging from 1=minimal engagement or rejection to 5=high involvement and expressivity.

**Table 1:** User engagement dimensions and rating scale.

Dimension and score			Description
**Verbal engagement**			
	1	Persistent silence or clearly disengaged verbal behavior (eg, hostile or rejecting remarks).	
	2	Minimal verbal output (eg, 1-word replies such as “yes” or “no”), delivered in a flat tone, with no spontaneous elaboration or follow-up.	
	3	Brief, functional responses that are appropriate and clearly articulated but emotionally neutral and lacking initiative.	
	4	Active participation through contextually appropriate answers and spontaneous follow-up contributions.	
	5	Fluent, sustained conversation featuring regular verbal initiative, coconstructed dialogue, and spontaneous, personally meaningful comments or anecdotes.	
**Physical engagement**			
	1	Defensive or avoidant posture (eg, crossed limbs and leaning away), evasive gaze, and rejecting or dismissive gestures.	
	2	Passive physical presence, with infrequent or minimal gestures and body oriented away from the robot.	
	3	Upright, neutral posture with limited physical expressivity; gaze may be directed toward the robot but lacks clear engagement; few gestures.	
	4	Open body orientation toward the robot, accompanied by expressive gestures and visibly responsive physical behavior (eg, nodding and leaning in).	
	5	Strong physical involvement, including spontaneous gestures, mimicry of the robot’s actions, or voluntary physical contact (eg, touching the robot).	
**Emotional engagement**			
	1	Clear negative emotional expression (eg, anger, frustration, and disgust), often accompanied by signs of interaction breakdown or withdrawal.	
	2	Very low emotional reactivity, flat facial affect, and visible signs of fatigue, boredom, or disinterest.	
	3	Neutral but attentive facial expression, stable gaze toward the robot, with limited or absent emotional expressivity.	
	4	Moderate social-affective signals, such as smiling, nodding, or short expressive reactions, indicating active but contained emotional engagement.	
	5	Strong affective display, including laughter, animated facial or physical expressions, and moments of emotional synchrony with the robot.	

The coding grid was designed by the research team, grounded in prior work on social signal processing [39], and iteratively refined through preliminary testing to ensure clarity, consistency, and applicability in naturalistic HRI settings. It incorporated both affective and interactional markers, such as speech spontaneity, posture alignment, gesture expressiveness, and emotional display.

All interactions were independently coded by 2 trained researchers (LB and MP) using a detailed coding manual to reduce subjectivity inherent in qualitative behavioral analysis [40]. In cases of disagreement, coders discussed the item to reach consensus; if consensus could not be achieved, a third researcher (JC) acted as an arbitrator to determine the final score. This multicoder approach aligns with best practices in HRI research, which emphasizes the use of multiple raters to mitigate individual bias and enhance intersubjective reliability [39,41].

Emotional engagement scores were not assigned for 28 of 130 interactions due to limited facial visibility caused by surgical masks, which impeded the reliable interpretation of affective expressions. These interactions involved 5 participants, 3 who remained masked throughout and 2 who removed their masks partway. While excluded from emotional engagement analysis, these sessions were retained for all other measures.

In addition to the quantitative ratings, coders were encouraged to document any ambiguous, unexpected, or contextually meaningful behaviors using an open comment field. These qualitative annotations served to refine and contextualize the interpretation of engagement patterns observed in the coded data.

#### Assessment Scales

Two standardized self-report questionnaires were administered immediately after the interaction to assess participants’ perceptions of the robot’s acceptability and usability. The Acceptability E-Scale (AES), adapted from Heerink et al [42] and translated into French by Micoulaud-Franchi et al [43], measures perceived acceptability across 6 dimensions: trust, perceived usefulness, enjoyment, sociability, ease of use, and intention to use. The scale includes 6 items, each rated on a 5-point Likert scale, yielding a total score ranging from 6 to 30. AES scores above 25.81 of 30 are considered to reflect high perceived acceptability, while lower scores indicate limited acceptability. The adapted version of the scale used in this study is provided in Multimedia Appendix 1.

The System Usability Scale (SUS) [44] is a widely used, validated 10-item questionnaire designed to assess perceived usability. Items are rated on a 5-point Likert scale, and the final score is calculated according to standard scoring procedures, yielding a composite usability score between 0 and 100. According to standard interpretive guidelines, SUS scores below 50.9 are considered poor, scores between 51 and 71.4 are rated as OK to good, and scores above 71.4 reflect excellent perceived usability [45]. The adapted version of the scale used in this study is provided in Multimedia Appendix 1.

#### Assessment Procedure

Each session (~45 minutes) followed a standardized sequence consisting of a short reminder of the objectives of the study, informed consent, interaction with the robot, and postinteraction assessments. Participants were first introduced to the robot’s general capabilities (eg, hospital orientation, entertainment, and appointment assistance) and informed that they could ask the robot questions freely related to these functions. No detailed interaction instructions or scripted prompts were provided, in order to preserve a naturalistic interaction context.

A fixed-position camera recorded the entire session for behavioral coding. A researcher (LB) was present throughout the entire session to provide assistance if needed, while minimizing interference.

Following the interaction, participants completed 2 standardized self-report questionnaires, the AES and the SUS, with support from the research team when necessary.

#### Data Analysis

Quantitative analyses were conducted using the jamovi software (version 2.4.11; The Jamovi Project) and focused on 4 dimensions: sociodemographic data, system performance, user engagement, and acceptability and usability.

For sociodemographic characteristics (age, sex, socioeducational level, and MMSE scores), descriptive statistics (mean, SD, minimum, and maximum) were first computed. Comparisons across experimental waves were then conducted using the Mann-Whitney *U* test, due to violations of normality and homogeneity of variance assumptions, as assessed by the Shapiro-Wilk and Levene tests.

For the analysis of HRIs, the unit of analysis was the individual interaction. Interaction duration was calculated for each HRI session based on video recordings. Mean durations were computed separately for each experimental wave, and the total cumulative duration of all recorded interactions was calculated to quantify the overall volume of video data analyzed. The definition of an interaction used in this study is provided in the System Performance section.

A descriptive analysis was first conducted for each wave, covering these elements: robot behavior categorized by 4 error types (comprehension failures, inappropriate verbal responses, incomplete responses, and technical errors) and the proportion of interactions with and without errors.

Subsequently, between-wave comparisons were performed. Robot behavior was compared across waves based on the average error rate per interaction. Analyses were first conducted considering all error types, followed by a focused analysis on the 3 verbal-related errors: comprehension failures, inappropriate verbal responses, and incomplete responses. These comparisons were conducted using Mann-Whitney *U* tests, due to nonnormal distributions and violations of homogeneity of variance in the error frequency data.

User engagement was then analyzed using multimodal behavioral coding across 3 dimensions: verbal, physical, and emotional engagement. A descriptive analysis was first conducted for each dimension. Subsequently, engagement scores were compared between experimental waves using Kruskal-Wallis tests, due to violations of normality and homogeneity of variance assumptions.

Interaction success was operationalized as the completion of a communicative goal, specifically, when the user successfully obtained the information requested from the robot. Success rates were compared between experimental waves using a chi-square test of independence to assess whether system upgrades influenced the likelihood of a successful exchange.

The dimensions of acceptability and usability, assessed using the AES and SUS questionnaires, respectively, were first examined through descriptive analysis. Subsequently, scores were compared between experimental waves using independent samples 2-tailed *t* tests, following confirmation of normality (Shapiro-Wilk test) and homogeneity of variance (Levene test).

To complement group-level comparisons, additional analyses were conducted to explore relationships between the dimensions of engagement, system performance, user experience, and participant characteristics. Pearson correlation analyses were used to examine associations between user engagement scores and interaction outcomes (ie, successful vs unsuccessful interactions) as well as between engagement scores and perceived acceptability (AES) and usability (SUS). Correlation analyses were also performed on the full sample to investigate relationships between sociodemographic characteristics (age, socioeducational level, and MMSE), system performance indicators (total and conversational error counts), user engagement dimensions (verbal, physical, and emotional), and subjective evaluations of the robot (AES and SUS). These analyses aimed to determine whether individual differences were associated with user experience, robot performance, or behavioral engagement during interactions.

A short qualitative analysis was conducted using thematic classification [46] of spontaneous, marginal comments made by participants during their interactions with the robot. These comments were not obtained through interviews or explicit discussions about the HRI but occurred naturally during the exchanges. Comments were categorized under emerging themes to complement the quantitative findings and provide additional insight into user engagement and interaction dynamics. Coding was performed independently by 2 observers (LB and MP), who then compared and discussed their categorizations to reach consensus on the final themes. Because of the small amount of data, no formal interrater reliability statistic (eg, Cohen κ) was calculated.

### Ethical Considerations

The study was approved by the French National Ethics Committee (“Comité de Protection des Personnes, CPP Ouest II [21.02.03.45524 2021/20], Maison de la Recherche Clinique – CHU Angers [2020-A02643-36]”; and complied with the General Data Protection Regulation. Data processing was registered with the Data Protection Officer (reference 20210114153645) in the Assistance Publique—Hôpitaux de Paris registry. The study did not involve randomization or a clinical intervention. Informed consent was obtained from all participants, and they were informed that they could withdraw from the study at any time. The original consent included approval for secondary analyses without requiring additional consent. All participant data were anonymized, and no compensation was provided. From an ethical standpoint, the use of SARs raises several issues, particularly regarding confidentiality and transparency. Because these systems collect and process sensitive data (vocal, visual, or interactional), their use and potential reuse must be explicitly stated. Ethical guidelines also demand that users and participants are clearly informed, so they understand how their data are managed. With the integration of LLMs, this obligation further includes clarifying whether experimental results will be used for model training and under which conditions.

## Results

### Participant Characteristics and Baseline Equivalence

The sample included 28 older adults, with 10 participants in wave 1 and 18 in wave 2. The mean age was 78.2 (SD 6.25; range 67-93) years, and most participants were women (n=20). The average MMSE score was 26.3 of 30 (SD 3.73; range 18-30), with scores ≥26 generally indicating normal cognitive function. The mean socioeducational level was 12.8 (SD 1.94) years, ranging from 9 (completion of lower secondary education or vocational training) to 14 or more years (university-level qualifications such as a bachelor’s degree or higher).

Baseline equivalence between groups was assessed using Mann-Whitney *U* tests for MMSE scores and socioeducational level, as both variables were nonnormally distributed. No significant differences were found between wave 1 and wave 2 for MMSE (*U*=49.5; *P*=.05; *r*=0.45) or socioeducational level (*U*=79.5; *P*=.57; *r*=0.12), supporting the comparability of the 2 experimental groups. The inclusion of effect sizes indicates that the observed differences were small to moderate in magnitude, further supporting baseline equivalence despite minor variability in MMSE. Participant characteristics by experimental wave are summarized in Table 2.

**Table 2:** Sociodemographic characteristics of participants by experimental wave.

Experimental wave	Sex		Age (years), mean (SD)	Education^a^ (years), mean (SD)	MMSE^b^ score, mean (SD)
	Male, n (%)	Female, n (%)			
Wave 1 (n=10)	3 (20)	7 (80)	78.6 (7.6)	13.1 (1.7)	28.0 (1.9)
Wave 2 (n=18)	6 (33.3)	12 (66.7)	77.9 (5.6)	12.6 (2.1)	25.3 (4.1)
Total (N=28)	9 (32.1)	19 (67.9)	78.2 (6.3)	12.8 (1.9)	26.3 (3.7)

^a^Years of education refer to the total years of formal schooling completed.

^b^MMSE: Mini-Mental State Examination (range 0-30); scores≥26 are generally considered within the normal cognitive range.

### Interaction Duration and Dataset Composition

**Figure 4:**
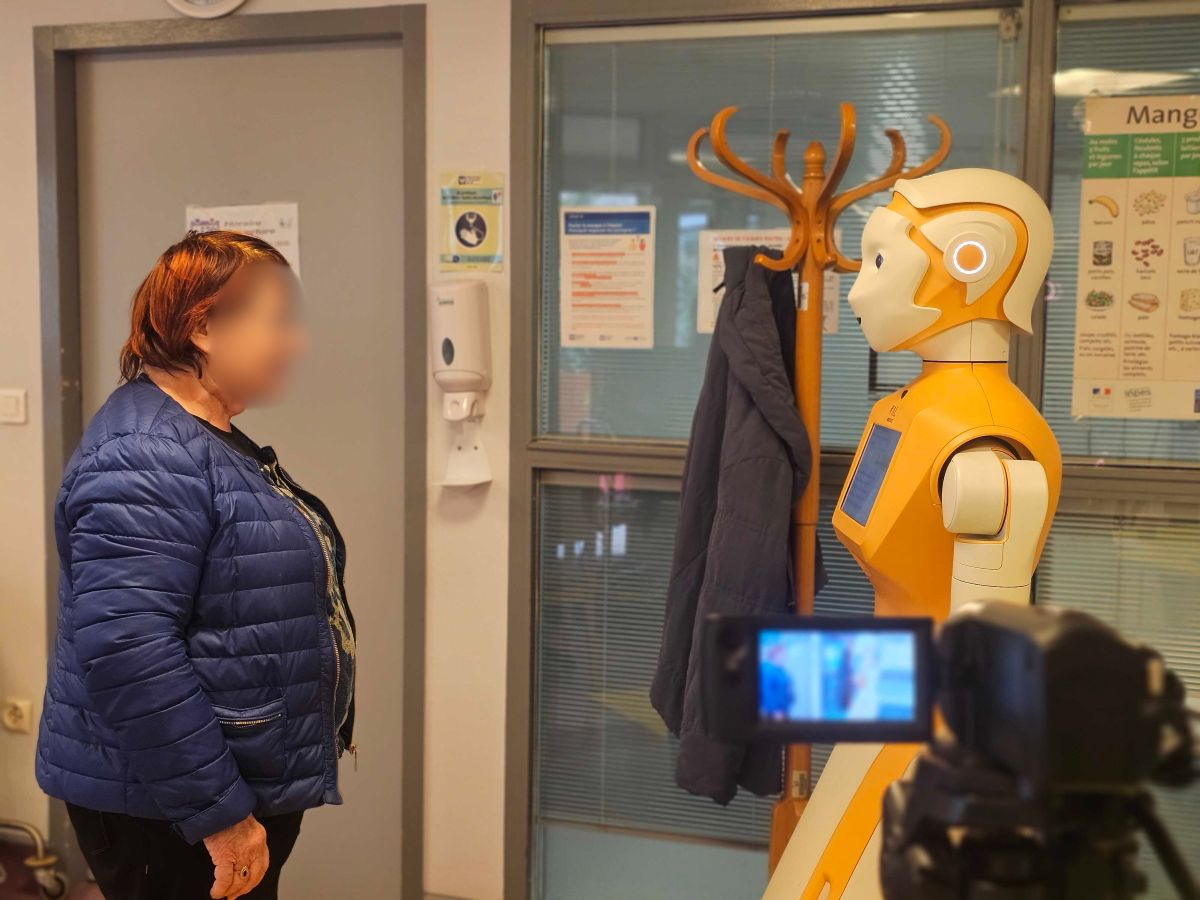
Experimental context: participant interacting with the socially assistive robot.

A total of 130 HRIs were analyzed (wave 1=36 HRIs and wave 2=94 HRIs), comprising the full dataset used for multimodal behavioral coding. Interaction durations ranged from 7 seconds to 3 minutes and 14 seconds, with a mean duration of 52 (SD 0.00038) seconds: wave 1=40 (SD 0.00037) seconds and wave 2=58 (SD 0.00037) seconds. In total, the recordings resulted in 1 hour, 53 minutes, and 55 seconds of video data. The experimental setup is shown in Figure 4, which depicts a participant interacting with the SAR during a session.

### Robot Error Patterns and System Performance Across Waves

#### Overview

Table 3 presents the distribution of robot error types across the 2 experimental waves. Comprehension failures were the most frequent error, occurring in 25.4% (33/130) of all interactions, with a notably higher rate in wave 1 (17/36, 47.2%) compared to wave 2 (16/94, 17%). Incomplete utterances were absent in wave 1 but appeared in 14.9% (14/94) of interactions in wave 2. Inappropriate verbal responses were also more common in wave 1 (13/36, 36.1%) than in wave 2 (14/94, 14.9%), contributing to a combined rate of 20.8% (27/130). Technical errors remained relatively stable between conditions, occurring in 11.1% (4/36) of interactions in wave 1 and 11.7% (11/94) in wave 2. For analysis, each error type was binary-coded per interaction (0=absent and 1=present).

**Table 3:** Distribution of robot error types by experimental wave.

Robots’ behaviors	Wave 1, n (%)	Wave 2, n (%)	Total, n (%)
Comprehension failures	17 (47.2)	16 (17)	33 (25.4)
Incomplete utterances	0 (0)	14 (14.9)	14 (10.8)
Inappropriate verbal responses	13 (36.1)	14 (14.9)	27 (20.8)
Technical error	4 (11.1)	11 (11.7)	15 (11.5)

Robot error types were compared across the 2 experimental waves based on total error counts. Verbal-related errors, including comprehension failures (*U*=989; *P*<.001; *r*=0.42), incomplete utterances (*U*=1440; *P*=.015; *r*=0.15), and inappropriate responses (*U*=1333; *P*=.008; *r*=0.21), differed significantly between wave 1 and wave 2. A higher number of verbal-related errors were recorded in wave 1, reflecting reduced conversational performance in this condition and suggesting improved system behavior following the updates implemented in wave 2.

An additional analysis was conducted at the level of individual interactions to compare error rates between experimental waves. Results showed higher error rates per interaction in wave 1 than in wave 2: comprehension failures occurred on average 2.03 times per interaction in wave 1 compared to 0.61 in wave 2; inappropriate responses occurred at a rate of 0.36 in wave 1 versus 0.15 in wave 2; and incomplete utterances, which were absent in wave 1, appeared with a mean frequency of 0.16 per interaction in wave 2. In contrast, technical error rates did not differ significantly between waves (*U*=1682; *P*=.93; *r*=0.01), indicating stable hardware performance across conditions.

Finally, the proportion of interactions without any system errors increased significantly in wave 2 following the introduction of the LLM (*U*=995; *P*<.001; *r*=0.41), indicating improved system stability and interaction reliability after the update. Only 27.8% (10/36) of interactions in wave 1 were error-free, compared to 70.2% (28/94) in wave 2. This substantial increase in error-free interactions suggests that the integration of the LLM contributed positively to the overall robustness and consistency of the system’s performance.

#### User Engagement

Participant engagement was evaluated using multimodal behavioral coding across 3 dimensions: verbal, physical, and emotional. Each dimension was scored on a 5-point scale, with higher scores indicating greater levels of engagement.

The mean verbal engagement score was 3.64 (SD 0.64) in wave 1 and 3.47 (SD 0.77) in wave 2. A Kruskal-Wallis test revealed no significant difference in verbal engagement between the 2 waves (*χ*^2^_1_=2.5; *P*=.11).

For physical engagement, scores remained stable across waves, with a mean of 3.03 (SD 0.77) in wave 1 and 2.99 (SD 0.73) in wave 2. No significant difference was observed (*χ*^2^_1_=0.06; *P*=.80).

Emotional engagement showed a slight increase, from a mean score of 2.92 (SD 1.02) in wave 1 to 3.33 (SD 0.86) in wave 2. No significant difference was observed (*χ*^2^_1_=3.4; *P*=.06). Descriptive statistics by wave are presented in Table 4.

**Table 4:** Comparison of acceptability and usability scores, user engagement metrics, and interaction success across waves.

Dimension	Wave 1	Wave 2	Difference, *P* value
AES^a^ score out of 30, mean (SD)	12.8 (4.58)	20.8 (6.52)	<.001
SUS^b^ score out of 100, mean (SD)	40.0 (24.04)	60.4 (23.11)	<.001
Duration of interaction (seconds), mean (SD)	40 (32)	58 (32)	<.001
Verbal engagement mean score out of 5 (SD)	3.64 (0.64)	3.47 (0.77)	.24
Physical engagement mean score out of 5 (SD)	3.03 (0.77)	2.99 (0.73)	.79
Emotional engagement mean score out of 5 (SD)	2.92 (1.02)	3.33 (0.86)	.05
Interaction success, n/N (%)	9/36 (25)	70/94 (74.5)	<.001

^a^AES: Acceptability E-Scale.

^b^SUS: System Usability Scale.

#### Interaction Success and Perceived Acceptability and Usability

Interaction success, defined as the completion of a communicative goal without breakdown, improved significantly across waves: 25% (9/36) in wave 1 versus 74.5% (70/94) in wave 2 (*χ*^2^_1_=26.7; *P*<.001).

Participants who experienced a successful interaction reported significantly higher ratings of both acceptability and usability. For the AES, mean scores were 21.6 of 30 (SD 5.84) in successful interactions, compared to 17.4 of 30 (SD 7.15) in failed ones (*U*=1261.5; *P*<.001; *r*=0.35). SUS scores followed the same pattern, with a mean of 63.3 of 100 (SD 22.5) for successful exchanges versus 51.1 of 100 (SD 26.5) for unsuccessful ones (*U*=1456.5; *P*=.01; *r*=0.25).

Importantly, no significant differences were found between participants who experienced successful versus unsuccessful interactions in terms of age (*U*=1898.5; *P*=.66; *r*=0.05), years of education (*U*=1915.5; *P*=.58; *r*=0.06), or MMSE scores (*U*=1921; *P*=.65; *r*=0.05), suggesting that interaction success was not dependent on participants’ demographic or cognitive profiles.

### Acceptability Assessment

**Figure 5:**
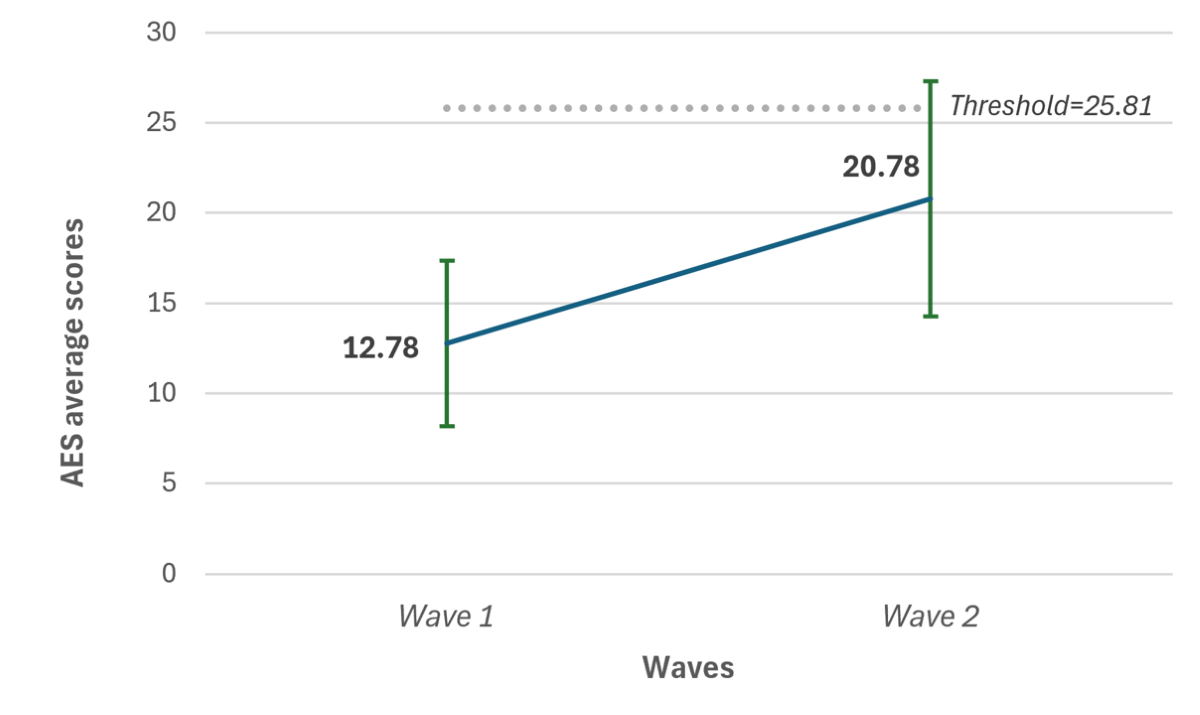
AES scores across the 2 experimental waves. AES: Acceptability E-Scale.

In wave 1, acceptability scores ranged from 9 to 23, with a mean of 12.8 (SD 4.58). In wave 2, scores ranged from 7 to 30, with a mean of 20.8 (SD 6.52). The AES ranges from 6 to 30, with higher scores indicating greater acceptability; a commonly used threshold of 25.81 denotes high acceptability. An independent sample 2-tailed *t* test revealed a significant difference between waves (t_25_=–3.28; *P*=.003), with higher acceptability observed in wave 2 compared to wave 1. Figure 5 illustrates the distribution of AES scores across the 2 experimental waves, showing the increase in mean acceptability from wave 1 to wave 2.

### Usability Assessment

#### Overview

**Figure 6:**
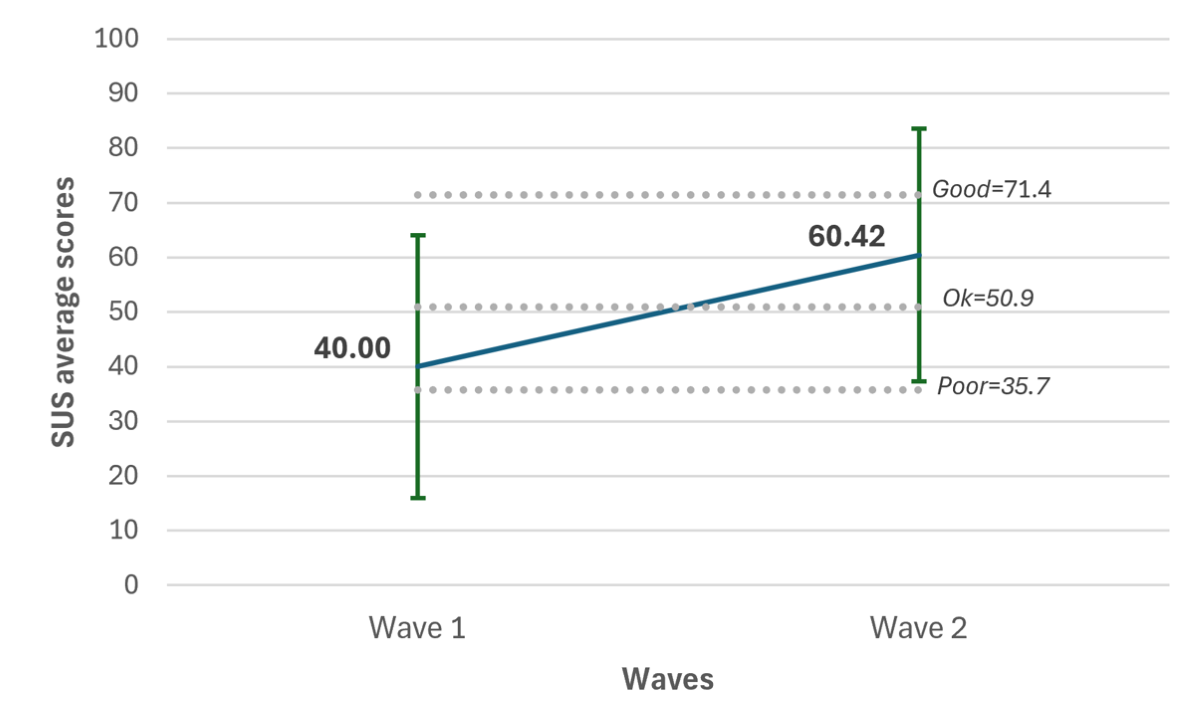
SUS scores across the 2 experimental waves. SUS: System Usability Scale.

In wave 1, SUS scores ranged from 12.5 to 67.5, with a mean of 40.00 (SD 24.04). In wave 2, scores ranged from 2.5 to 92.5, with a mean of 60.42 (SD 23.11). The SUS ranges from 0 to 100, with 50.9 commonly cited as the minimum threshold for acceptable usability; scores below this indicate insufficient usability. An independent sample 2-tailed *t* test revealed a significant difference between waves (t_25_=–2.14; *P*=.04), indicating greater perceived usability in wave 2 compared to wave 1. Figure 6 shows the increase in mean SUS scores between the 2 experimental waves, indicating improved perceived usability in wave 2.

#### Associations Among Engagement, Outcomes, and User Characteristics

A Pearson correlation analysis was conducted to explore the relationships between user engagement (verbal, physical, and emotional), interaction outcomes, system evaluations, and participant characteristics (see Multimedia Appendix 2 for the full correlation matrix). Emotional and physical engagement were strongly positively correlated (*r*=0.63; *P*<.001), indicating that increased emotional expressiveness was closely associated with greater physical involvement. Emotional engagement was also positively associated with interaction success (*r*=0.28; *P*=.008), suggesting that successful interactions tended to elicit higher emotional responsiveness.

A moderate positive correlation was observed between physical and verbal engagement (*r*=0.23; *P*=.009), indicating that participants who were more physically expressive also tended to engage in more sustained or contextually responsive verbal behavior. Conversely, verbal engagement was negatively correlated with perceived acceptability (AES; *r*=–0.27; *P*=.002), suggesting that more frequent or proactive verbal contributions were associated with lower acceptability ratings.

Among participant characteristics, age showed a moderate negative correlation with physical engagement (*r*=–0.30; *P*<.001), indicating that older participants tended to be less physically expressive during interactions. Age was also negatively associated with acceptability ratings (*r*=–0.20; *P*=.03), suggesting slightly reduced system acceptability among older users. No significant correlations were found between cognitive functioning, as measured by MMSE scores, or socioeducational level, and the other engagement or evaluation measures.

#### Qualitative Analysis of Spontaneous Verbalizations

#### Overview

During the interactions, participants occasionally produced spontaneous comments in reaction to specific events, often triggered by the robot’s conversational or technical errors. These remarks provide insight into how older adults evaluated the exchange, revealing patterns of frustration, adaptation, humor, and expectation management. Thematic analysis identified 5 recurrent themes (Table 5).

**Table 5:** Thematic analysis of participants’ spontaneous verbalizations.

Theme	Description	Exemplar quotes
Silence, confusion, and humor as coping	Errors or nonresponses from the robot often led participants to react with humor, irony, or playful comments, softening frustration.	“We’re going round in circles here. [laughs]” [Wave 1]. “Does it [the robot] have blocked ears? [laughs]” [Wave 1). “Yet [the robot] understood (points to the screen with the transcript) but it [the robot] doesn’t answer my question” [Wave 1].
Self-attribution of blame	Communication breakdowns were sometimes attributed by participants to their own speech, hearing, or contextual factors.	“I can’t hear very well, as I have a lot of trouble with my ears” [Wave 1]. “It [the robot] is having trouble understanding me. Is it the mask?” [Wave 2]. “Am I speaking loud enough [for the robot to understand]?” [Wave 1].
Expectation-response mismatch and technology comparisons	Participants noted discrepancies between expected and actual robot responses, sometimes comparing them to earlier or limited technologies.	“Doesn’t it understand anything? [the robot did not give an accurate answer]” [Wave 1]. “We’re not going to upset it [laughs]” [Wave 1]. “It reminds me of when they first introduced voice word processing. We had some absolutely incredible things written down. It didn’t make any sense at all” [Wave 2].
Seeking external guidance	Interaction difficulties prompted participants to turn to the experimenter for clarification or support.	“How can I find out what questions I can ask the robot? [to the experimenter]” [Wave 2]. “Do I have to talk to it [the robot]?” [Wave 2]. “So now I can start again? Do I have to restart from zero or should I step out of the field and come back? [to the experimenter]” [Wave 2].
Positive reactions to robot-initiated exchanges	When the robot took initiative, participants expressed surprise or delight, highlighting novelty and engagement.	“It’s impressive [the robot]!” [Wave 2]. “Oh! It [the robot] talks to me first, just like a person!” [Wave 2]. “So if I don’t say anything, it [the robot] will keep asking me things?” [Wave 2].

#### Silence, Confusion, and Humor as Coping

When the robot failed to respond or gave irrelevant answers, participants often reacted with a mix of confusion and humor. Some expressed mild frustration, “Well, yes, but it’s not answering me” (Wave 1), while others turned to playful remarks, joking that the robot had “blocked ears” or even deserved a slap. Sarcasm and irony also emerged as ways to reframe disappointment, with comments like “[laughs] Wasn’t that question planned [in the database]?” (Wave 1) or “We asked it a question it’s not very good at” (Wave 1). Participants also relied on the robot’s transcript display to make their point: “It understood (points to the screen) but it doesn’t answer my question.” These responses show how older adults shifted between frustration and collaborative problem-solving. Some participants therefore chose humor rather than disengagement, demonstrating resilience when interacting with a new technology such as SAR.

In addition, participants recurrently described conversational “loops” when repeated prompts or ARI’s lack of uptake brought dialogue to a standstill. The interaction frequently felt circular: the robot kept repeating information and failed to build on prior exchanges. This resulted in participants asking clarifying questions like “Shall I continue?” (Wave 2), signaling a breakdown in mutual understanding. Affect often shifted from amused to impatient, captured in remarks such as “We’re going round in circles here [laughs]” (Wave 1) and “Wake up ARI!” (Wave 1), yielding interactional impasses marked by circularity, uncertainty about mutual understanding, and mounting frustration.

#### Self-Attribution of Blame

Instead of attributing breakdowns to the robot’s limitations, participants often questioned their own role in communication difficulties. Comments such as “I can’t hear very well, as I have a lot of trouble with my ears” (Wave 1) or “It’s having trouble understanding me. Is it the mask?” (Wave 2) show how challenges were internalized, sometimes linked to environmental factors or speech clarity. This self-scrutiny extended to interaction etiquette, “Do I speak fast enough?” (Wave 1), “Am I speaking loud enough?” (Wave 1), and even basic rules, as in “Ah! Because I have to ask a question?” (Wave 2). Such attributions highlight how older adults often personalize technological breakdowns, with uncertainty toward novel systems manifesting as self-questioning rather than solely blaming the robot.

#### Expectation-Response Mismatch and Technology Comparisons

Participants at times noted a gap between their expectations and the robot’s actual capabilities. Remarks such as “It’s a catering robot, not a robot for guiding patients” or “Doesn’t it [the robot] understand anything?” (Wave 1) revealed disappointment or confusion about its function. Others responded more playfully or with emotional distance, “We’re not going to upset it [the robot] [laughs]” and “It [the robot] looks at me with strange eyes” (Wave 1). Some also drew comparisons with earlier technologies, as in “It [the robot] reminds me of when they first introduced voice word processing. We had some absolutely incredible things written down, it didn’t make any sense at all” (Wave 2). Such expressions reflect a mix of critique and humor, showing how unmet expectations often evoked both skepticism and familiarity with past technological shortcomings.

#### Seeking External Guidance

When conversational flow was disrupted or when uncertainties about the interaction emerged, several participants turned to the experimenter for clarification or support. Requests like “Come along please [to the experimenter]” (Wave 2) and “How can I find out what questions I can ask the robot?” (Wave 2) point to participants’ desire for guidance on possible actions or system boundaries. This tendency was also evident in more basic queries, “Do I have to talk to it [the robot]?” and logistical questions, for example, “So now I can start again? Do I have to restart from zero or should I step out of the field and come back? [to the experimenter]” (Wave 2). These excerpts highlight not only a search for reassurance but also the need for clearer cues and support mechanisms within the human-robot interface, especially in scenarios where expectations and interaction rules may not be fully explicit.

#### Positive Reactions to Robot-Initiated Exchanges

Participants sometimes reacted with surprise or curiosity when the robot initiated the conversation. Exclamations such as “It’s impressive! [the robot]” (Wave 2) or “Oh! It [the robot] talks to me first, just like a person!” (Wave 2) show an appreciation for its apparent autonomy and proactive engagement. This behavior was perceived as a sense of dynamism to the interaction. The realization that the robot could sustain the exchange on its own, “So if I don’t say anything, it will keep asking me things?” (Wave 2), further stimulated engagement.

## Discussion

### Principal Findings

This study evaluated the integration of a SAR into the routine activities of a geriatric hospital unit through a 2-wave, real-world comparative design. In both waves, older adults interacted freely with the robot within its functional domains (eg, hospital orientation, entertainment, and appointment assistance), without scripted prompts, in order to preserve the spontaneity. Quantitative measures included system performance indicators (error types and rates), user engagement (verbal, physical, and emotional dimensions), interaction success, and subjective evaluations of acceptability and usability; these were complemented by a thematic analysis of postinteraction interviews focusing on perceptions of robot errors.

Results showed that the introduction of an LLM between waves was associated with marked improvements in conversational performance, including a substantial reduction in verbal errors, an increase in error-free interactions, and higher rates of task success. These gains in system reliability were mirrored by higher acceptability (AES) and usability (SUS) ratings in the second wave. Yet, these gains, concerning HRI and user experience, should not be viewed as solely the product of the LLM; aspects of the study design (eg, different samples) and contextual factors (eg, the kind of questions users asked the robot in each wave) may also have shaped the outcomes.

On another level, qualitative analysis showed that participants’ spontaneous reactions to the robot were shaped by both system performance and their own expectations. Breakdowns often prompted humor, self-blame, or requests for clarification, while successful or unexpected robot-initiated exchanges could elicit surprise and positive engagement. These findings suggest that emotional and behavioral responses in SAR-older adult interactions are influenced as much by expectation management and novelty by technical reliability.

These observations are consistent with prior work on how humans perceive and respond to erroneous robots. Mirnig et al [47] found that user reactions to robot errors are not exclusively negative but often include adaptive strategies such as humor, indulgence, or self-attribution of blame. Minor errors may even strengthen engagement by rendering the robot more “human-like,” provided that such errors remain occasional and do not critically hinder task achievement. In line with this, several of our participants coped with breakdowns through irony or laughter, suggesting that, in geriatric care contexts, error management is not only a technical challenge but also a social and affective process shaping the overall interaction climate.

The following discussion interprets these findings in light of existing literature, examining how the observed changes in performance, engagement, and user perceptions contribute to current knowledge on SAR deployment in geriatric care and highlighting implications for future system design and implementation.

### LLM Integration and Conversational Reliability

The results suggest that the integration of the LLM, introduced between wave 1 (without LLM) and wave 2 (with LLM), enhanced the SAR’s ability to process diverse user input and produce contextually relevant responses. This improvement was evident not only in lower rates of comprehension failures and incoherent replies but also in greater interactional fluidity, characteristics essential for sustaining user engagement. The marked improvement in interaction success across waves (wave 1: 9/36, 25% vs wave 2: 70/94, 74.5%) further underscores the practical benefits of LLM integration for enabling communicative goals to be achieved without breakdowns. These gains align with the observed reductions in verbal errors and the higher proportion of error-free interactions, indicating that technical reliability is closely tied to the system’s capacity to support smooth exchanges. In addition, successful interactions were associated with higher acceptability (AES) and usability (SUS) ratings, reinforcing prior evidence that perceived system competence is a key determinant of user satisfaction and trust in SARs.

Such outcomes align with initial evidence from other domains, including customer service [48], museum guidance [49], and the use of SARs for education [50,51], where LLMs have been reported to improve language comprehension, contextual adaptation, and response coherence [52]. Compared to rule-based or task-specific systems, systems equipped with LLM seem to offer more fluid, adaptive, and context-sensitive interactions [53,54], which is consistent with our findings, showing a significant improvement in interaction success and perceived acceptability and usability after LLM integration.

Recent work with older adults further demonstrates the potential of LLM-powered systems to support health and well-being in real-life care contexts. For example, conversational agents have been used in home environments to monitor safety risks, verify symptoms, and initiate alerts during emergencies [55]. Other studies have leveraged LLMs to collect richer health information with less provider effort [56] or to deliver cognitive stimulation through structured dialogue, resulting in improved task performance, social engagement, and high acceptance among older adults [57].

### System and User Constraints in LLM-Enhanced Older Adult-Robot Interaction

However, LLM-based applications, whether implemented in clinical or everyday settings involving older adults, also reveal limitations that can arise from both the system and the use. On the system side, latency from cloud-based speech recognition and LLM processing, as observed in Lima et al [57], can interrupt conversational flow and disrupt turn-taking, sometimes being perceived as inattentiveness. These challenges are consistent with broader observations in spoken dialogue systems for robotics, where developers must balance trade-offs between accuracy and latency [58]. While cloud-based systems often deliver higher accuracy, they introduce delays that can break the rhythm of interaction; conversely, on-premises systems avoid latency but are typically less accurate and more limited in vocabulary. Furthermore, pretrained recognizers are usually optimized on datasets that differ from the spontaneous, fragmentary, and context-dependent speech, common in real-world robot use, making adaptation resource-intensive. In our study, similar conversational breakdowns occurred in verbal-related errors, such as incomplete, irrelevant, or incoherent responses, which interrupted the exchange and occasionally led to repeated, circular interactions. We believe this might be due to a combination of several factors. First, even if we adapted the speech recognition model with French data, our on-premises solution has its limitations in terms of accuracy and confuses some words. Second, even if the LLM solution operated on a partner’s cloud with sufficient resources, it could not always provide accurate and appropriate answers, even when the speech recognition worked flawlessly. Third, the system used a half-duplex audio, listening to the user only when the system was not speaking, which reduced interaction fluidity. Finally, even if these limitations are mild individually, their combination could cause misinterpretation on the robot’s side and generate user frustration, which did not ease the next steps of the interaction.

A further system-related limitation is that current LLMs are not specifically trained for interactions with older adults. As noted by Diaz et al [59] and Chu et al [60], speech and interaction data from older adults are scarce in artificial intelligence (AI) training corpora, and existing datasets often contain age-related biases. As a result, characteristic features of older adult communication, such as changes in prosody, vocabulary, conversational pacing, and the presence of hesitations or fragmented discourse in noisy environments, are underrepresented. This underrepresentation may reduce the ability of LLM-based systems to accurately interpret older adult speech, anticipate their communicative needs, and adapt to their interaction style in real-world settings.

From the user perspective, common age-related factors such as slower speech tempo, pauses, and fluctuations in volume can pose difficulties for speech recognition, particularly in the presence of cognitive decline [61]. Beyond these physiological and cognitive aspects, there is also an expectation gap in how older adults perceive conversational systems. Mahmood et al [62] describe how older adults often approach such technologies through the lens of human conversational norms, anticipating richer and more contextually adaptive exchanges than the system can actually deliver. This tendency is reinforced, as noted by Mahmood et al [63] and Liu et al [64], by two recurring influences: (1) the way many voice interfaces are designed and promoted to mirror human conversational styles and (2) the inherent affordance of speech as an interaction modality, which can implicitly suggest that open-ended, human-like dialogue is possible when, in practice, the system’s scope is more constrained and task-oriented.

These perceptions can be resistant to change, even with repeated exposure, and may lead to frustration when the robot’s responses fall short of these implicit promises [65,66]. In our study, this was evident in qualitative data: some participants persisted in treating the robot as a human interlocutor, even after repeated conversational errors. For example, one participant humorously asked, “Does it [the robot] have blocked ears?” (Wave 1), while another, after receiving no reply, remarked, “Well, yes, but it [the robot] is not answering me” (Wave 1). Others attributed breakdowns to their own performance, asking, “Am I speaking loud enough [for the robot to understand]?” (Wave 1) or “Do I speak fast enough [for the robot to understand]?” (Wave 1). In some cases, repeated failures prompted disengagement or the need for external guidance, as illustrated by the query, “How can I find out what questions I can ask the robot?” (Wave 2).

### User Engagement and Its Relationship With Interaction Outcomes

Verbal, physical, and emotional engagement scores in our study remained relatively stable between waves, with no statistically significant differences. Although wave 2 showed an improvement in interaction success, acceptability, and usability, these gains did not directly translate into measurable increases in engagement behaviors.

Emotional engagement showed a modest upward trend from wave 1 to wave 2, potentially reflecting more fluid exchanges with the LLM-enhanced system, but this change did not reach statistical significance. Because participants in the 2 waves were different individuals, the pattern is unlikely to reflect individual-level adaptation over time and may instead be related to situational factors or differences in interaction dynamics. It is also possible that more pronounced differences in engagement were not detected because the observed interactions were brief (approximately 52 seconds on average) or because the 5-point coding scale used to assess engagement may not have been sufficiently sensitive to capture subtle variations. Moreover, many of the analyzed exchanges were primarily functional in nature, where high levels of observable emotional engagement would not necessarily be expected, even successful interactions can occur with minimal emotional display (eg, “Where are the toilets?” (User), “At the end of the corridor, on your left” (Robot)).

Our findings also revealed strong correlations between emotional and physical engagement, indicating that when users are emotionally responsive, they also tend to be more physically or behaviorally expressive. However, unlike system performance indicators, these engagement dimensions were not directly associated with interaction success. For instance, participants sometimes laughed or joked at robot errors.

Interestingly, verbal engagement was negatively correlated with acceptability, echoing findings by Mahmood et al [63] that higher verbal activity in older adult-robot interactions can sometimes stem from the need to overcome conversational breakdowns rather than from enjoyment. In our context, this may indicate that some users spoke more in an attempt to repair misunderstandings, potentially lowering their subjective evaluations of the system.

### Accessibility Challenges in Multimodal Robot-Older Adult Interaction

Across conversational HRI with SARs in geriatrics, particularly those powered by AI-based dialogue models, key accessibility concerns span model-training quality (representativeness and bias), user diversity and inclusion (cognitive, linguistic, sensory, and cultural variability), and interface ergonomics (voice or visual or physical modalities and interaction load).

First, studies highlight the underrepresentation of older adults in generative AI training databases, leading to persistent generational, ethnic, and cultural biases and limiting the recognition of nicknames, idioms, or nonstandard expressions in HRI [67-70].

Second, age-related changes in voice, such as reduced intelligibility, rhythm fluctuations, or speech disorders (dysarthria and aphasia), further complicate dialogue quality and voice processing [71,72]. Although targeted adaptation of acoustic models can reduce error rates, most recognition systems remain unable to accurately integrate the modulation and heterogeneity of vocal productions related to age and pathologies [73].

Third, in terms of interfaces, using the ARI tablet to display transcripts is a way of compensating for hearing or cognitive impairments, but literature on sensory impairment shows that true accessibility requires multimodal support (visual, tactile, and auditory). This includes optimized font size and contrast, pictograms, and gestural instructions for different levels of visual impairment [74-76].

Finally, the study did not assess the impact of specific pathologies (eg, aphasia and Parkinson disease) on speech recognition or multimodal adaptation. Future SAR development should rely on more diverse datasets, advanced multimodal calibration, and adaptive features (volume, articulation, and gesture sensitivity) to enhance inclusion and responsiveness in geriatric care.

### Limitations of the Study

This study has several limitations. First, it was conducted in a single geriatric hospital unit, which limits the generalizability of the findings to other care contexts or to community-dwelling older adults. The relatively small sample size may have reduced the statistical power to detect certain associations, particularly regarding user characteristics. In addition, the 2-wave, cross-sectional design, where different participants were recruited for each wave, limits our ability to isolate the impact of the LLM from potential differences in participant profiles. Because the individuals differed between samples, variations in their characteristics may partly explain the observed differences in HRI quality. A within-subjects design would allow a more precise assessment of the LLM’s specific contribution to the outcomes.

Second, this study focused on stationary, face-to-face interactions and did not explore the effect of robot mobility (eg, autonomous navigation) or expressive gestures on user engagement. Prior research has shown that these dimensions can significantly enhance social presence and interactional involvement in SAR-older adult encounters, particularly in stimulating physical engagement and sustained attention [30].

Third, the analysis focused on short-term, functional interactions. It did not capture the relational and identity-based dimensions of conversation, which are especially meaningful for older adults. For many older adults, dialogue is not only just about obtaining information but also about sustaining social ties and sharing narratives of lived experiences. By overlooking these deeper aspects, our findings may underestimate key factors influencing long-term engagement with SARs. Future work should explore how LLM-enhanced dialogue systems can use biographical cues, narrative prompts, and adaptive strategies to better reflect the relational nature of older adults’ communication.

Finally, the short interaction duration (52 seconds) provided only a snapshot of spontaneous use, limiting conclusions about long-term engagement and sustained acceptance. Longitudinal deployments are needed to capture how perceptions, trust, and interaction patterns evolve over extended use.

### Recommendations for Future Development and Implementation of Conversational SARs Using LLMs

This study highlights the potential of conversational SARs powered by LLMs to substantially improve conversational reliability with older adults. Future development should build on these gains while addressing key limitations and contextual needs observed in our findings.

#### Adapt AI Architectures for Older Adult Use, Including Future Vision-Language Model and Vision-Language-Action Model Applications

Beyond LLMs, future conversational SARs are likely to incorporate vision-language models and vision-language-action models, enabling richer multimodal understanding and more context-aware behaviors. However, all such models should be specifically adapted for older adults, particularly those with cognitive impairment, whose speech patterns, vocabulary, cognitive processing, and sensory capabilities differ from the datasets typically used to train general-purpose systems. Responsible adaptation must comply with regulatory and ethical frameworks to ensure safe, equitable, and trustworthy use in care settings.

#### Address the Limited Prior Experience of Older Adults With Conversational SARs Through Structured Familiarization Periods

Many older adults, particularly those without prior exposure to conversational agents, may require time and guided support to understand the robot’s capabilities, limitations, and “rules of engagement.” Future implementations should include a facilitator-led familiarization phase, where older adults can safely explore functions, interaction styles, and question formats without performance pressure. This early stage can build confidence, reduce uncertainty, and lay the groundwork for more meaningful and sustained engagement once independent use begins.

#### Strengthen the Facilitator’s Role During Familiarization

For both novice and older adults with cognitive impairment, a human facilitator can play a critical role in bridging early HRI challenges. This includes demonstrating effective input styles, explaining the robot’s capabilities and limitations, and supporting older adults during breakdowns. Incorporating a structured familiarization period may accelerate learning, reduce frustration, and foster more positive first impressions.

#### Implement Adaptive Error-Handling Strategies

Conversational SARs should detect and repair interaction breakdowns early, such as prolonged silences, repeated input, or off-topic responses, by offering clarifying prompts, rephrased questions, or multimodal alternatives (eg, visual selection and touch inputs). This can maintain engagement and reduce the negative emotional impact of errors.

#### Enhance HRI Personalization Across Sensory, Cognitive, and Motivational Dimensions

Personalization should include tuning speech recognition thresholds, adjusting interaction pacing, adapting vocabulary complexity, and aligning content with individual preferences and abilities. For older adults with varying cognitive, sensory, and mobility profiles, this is critical for both accessibility and engagement.

#### Integrate Proactive and Context-Aware Conversational Strategies

Initiative-taking behaviors, such as relevant follow-up questions, personalized activity suggestions, and reference to prior interactions, can help sustain older adult interest. Care should be taken to ensure that such behaviors remain contextually appropriate and nonintrusive.

#### Evaluate Future Systems With Multidimensional Frameworks

Assessment should combine technical measures (eg, error rates and latency), behavioral indicators (eg, engagement scores and turn-taking dynamics), and experiential outcomes (eg, trust and perceived usefulness). Such an approach ensures that improvements target both system performance and user experience in real-world contexts.

#### Promote Secure and Transparent Deployment

Beyond technical performance, deploying LLM-enhanced SARs in geriatric care requires attention to ethics and practice. In our study, only consenting participants were recorded, and all data were pseudonymized and securely stored in compliance with the General Data Protection Regulation. While these measures reduce privacy risks, future use will demand strict safeguards and transparency on secondary data use. Caution is also needed against overtrust, as older adults may attribute undue authority to robots. Clear system limits, caregiver oversight, staff training, and assessment of costs are vital to ensure safe, equitable, and sustainable integration in care.

### Conclusions

In this real-world, 2-wave study in a geriatric hospital day care unit, integrating an LLM into a conversational SAR improved interaction success, conversation quality, and perceived system performance. Comparing a baseline dialogue system (wave 1) with an LLM-enhanced system (wave 2) showed that advanced AI can produce more coherent, contextually relevant exchanges. These improvements were reflected in higher acceptability and usability ratings from older adults. Conducting the study in a naturalistic care environment was key to capturing spontaneous interactions and real-world constraints, factors often absent in laboratory settings.

Our findings confirm the potential of LLMs to enhance HRI in care contexts. However, they also highlight the need to better understand how interaction success relates to engagement quality and to user sociodemographic characteristics. To maximize these benefits, future systems should be tuned to older adults’ speech, cognitive, and sensory profiles. Strategies such as structured familiarization periods, adaptive error recovery, and personalized interactions could help maintain engagement over time. Aligning technical capabilities with the needs and preferences of older adults will be essential for making conversational SARs trusted and effective tools for geriatric care.

## Appendix

Multimedia Appendix 1 Evaluation scale Acceptability E-Scale and System Usability Scale (translated from French).

Multimedia Appendix 2 Correlations among behavioral, successful, and subjective measures.

## Abbreviations

AES: Acceptability E-Scale
AI: artificial intelligence
DCH: day care hospital
HRI: human-robot interaction
LLM: large language model
MMSE: Mini-Mental State Examination
SAR: socially assistive robot
SPRING: Socially Pertinent Robots in Gerontological Healthcare
SUS: System Usability Scale

## Data Availability

The datasets generated or analyzed during this study are available from the corresponding author on reasonable request.
