# Supplementary material for: Integrating a Large Language Model Into a Socially Assistive Robot in a Hospital Geriatric Unit: Two-Wave Comparative Study on Performance, Engagement, and User Perceptions
Source: JMIR Hum Factors. 2025 Dec 3;12:e81936. doi: 10.2196/81936 (PMC12712570; doi:10.2196/81936)
Supplement: Multimedia Appendix 1 [file humanfactors_v12i1e81936_app1.docx]

- *5-point Likert scale*

**Acceptability E-scale (AES)**

1. How easy did you find this robot to use?
2. How understandable were the robot's response and suggestions?
3. How much did you enjoy using this robot?
4. How useful was this robot in answering your questions?
5. Was the time taken by this robot to answer your questions acceptable?
6. How would you rate your general satisfaction with this robot?

**System Usability Scale (SUS)**

1. I would like to use (talk to, interact with) this robot in the hospital as often as possible.
2. I find conversations with this robot unnecessarily complex.
3. I think this robot is easy to use (talk to, interact with, operate).
4. I think I will need help to be able to interact (talk to, communicate with) this robot.
5. I found that the various services (functions) of this robot were well thought out (designed).
6. I think there are too many inconsistencies with this robot.
7. I imagine that most people would be able to learn how to use this robot very quickly.
8. I found it very difficult to talk and behave naturally with this robot.
9. I felt very confident using the robot.
10. I think there is a lot to learn in order to use this robot properly (talking, behaving, acting).
