# Supplementary figures and images for: Integrating a Large Language Model Into a Socially Assistive Robot in a Hospital Geriatric Unit: Two-Wave Comparative Study on Performance, Engagement, and User Perceptions
Source: JMIR Hum Factors. 2025 Dec 3;12:e81936. doi: 10.2196/81936 (PMC12712570; doi:10.2196/81936)

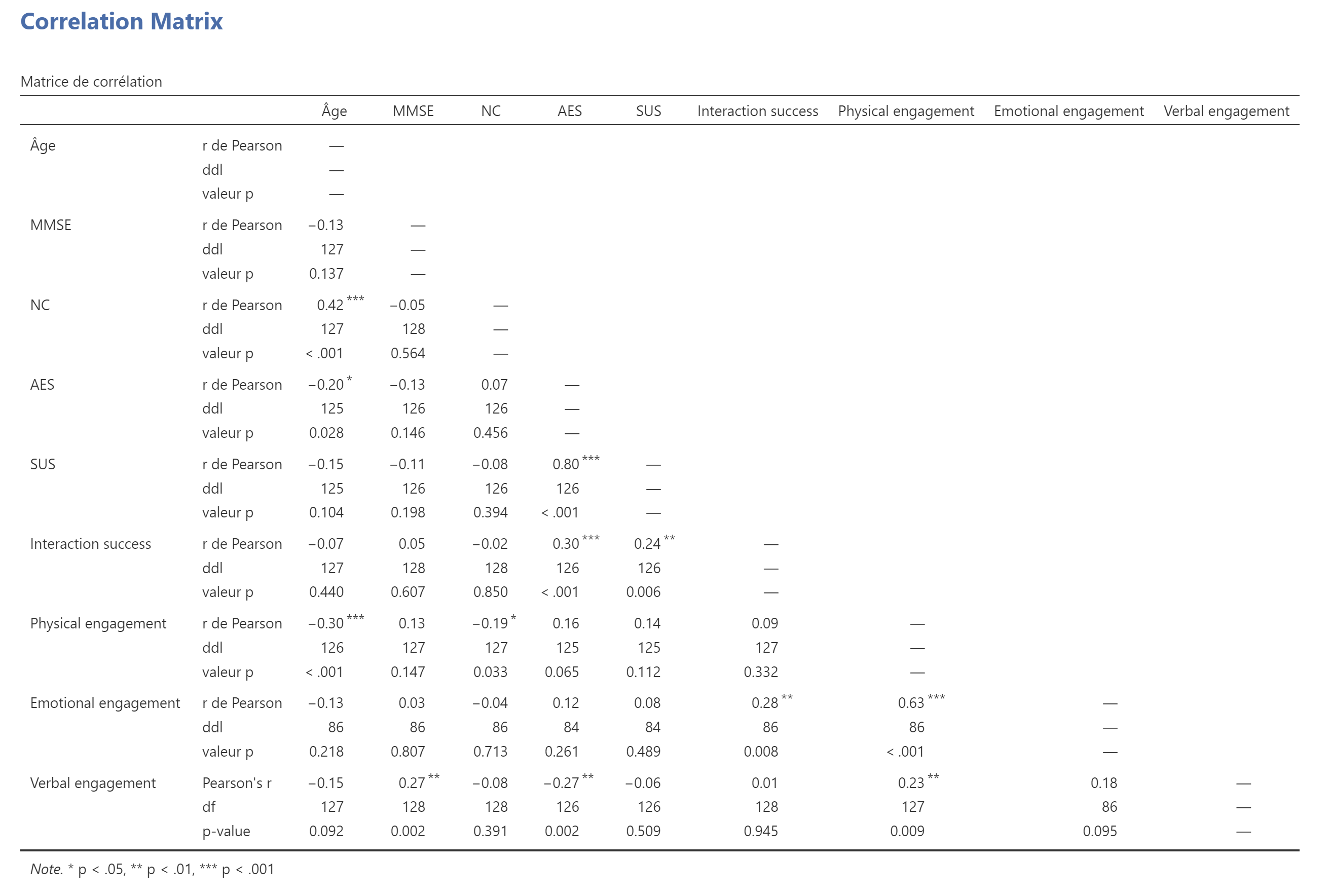

Supplement: Multimedia Appendix 2 [file humanfactors_v12i1e81936_app2.docx]
